# The Effects of Cervical Manipulation Compared with a Conventional Physiotherapy Program for Patients with Acute Whiplash Injury: A Randomized Controlled Trial

**DOI:** 10.3390/healthcare13070710

**Published:** 2025-03-24

**Authors:** Joan Parera-Turull, Maite Garolera, Jose-Blas Navarro, Dolors Esteve Bech-Decareda, Josep Gual-Beltran, Jose-Vicente Toledo-Marhuenda, Emilio-Jose Poveda-Pagan

**Affiliations:** 1 Clinical Research Centre d’Osteopatia Terrassa, Consorci Sanitari de Terrassa, 08221 Terrassa, Spain; joanparera@osteopatiaterrassa.com; 2 Clinical Research Group for Brain, Cognition and Behavior, Consorci Sanitari de Terrassa, 08221 Terrassa, Spain; mgarolera@cst.cat (M.G.); lesteba@cst.cat (D.E.B.-D.); jgualb@cst.cat (J.G.-B.); 3 Department of Psychobiology and Methodology of the Health Sciences, Universitat Autònoma de Barcelona, Bellaterra, 08193 Barcelona, Spain; joseblas.navarro@uab.cat; 4 Center of Translational Research in Physiotherapy, Department of Pathology-Surgery, Physiotherapy Area, Faculty of Medicine, Miguel Hernández University, 03550 Sant Joan d’Alacant, Spain; ejpoveda@umh.es

**Keywords:** whiplash injuries, manipulation, manual therapy, physical therapy, exercises, neck pain

## Abstract

Whiplash injuries (WLs) are the most frequent cause of emergency room visits after motor vehicle collisions. In clinical practice, massage, electrotherapy, mobilization, or therapeutic exercise are used. As part of manual therapy, high-velocity, low-amplitude manipulative techniques can also be used. **Objectives**: To evaluate the effect of the cervical Specific Adjustment Technique (SAT) in adults affected by whiplash on pain, functionality, cervical mobility, and radiological changes in cervical curvature through a prospective, single-blind, randomized clinical trial. **Methods**: One hundred and nineteen patients with grade II acute WL were randomly assigned to either the manipulation group (MAN group = 59) or the rehabilitation group (RHB group = 60) to receive 3 or 20 sessions of treatment, respectively. Both groups were measured at baseline and 15, 30, and 120 days after starting treatment. **Results**: Statistically significant differences were found in the MAN group in flexion (*p* = 0.041) and left-side bending (*p* = 0.022); similar statistical values were found in the other measures. According to the interaction treatment-time effect, statistical significance for the Cobb angle was obtained in the MAN group (*p* = 0.047). **Conclusions**: the effects of SAT were comparable in terms of pain, functionality, and mobility of the cervical spine. Although further research is needed on its effects in the acute phase, due to its effectiveness and lower associated cost, SAT could be considered a useful technique, at least during the first 3 months after a traffic collision.

## 1. Introduction

Whiplash injuries (WLs) and their consequences, along with whiplash-associated disorders (WADs) [1], are the most frequent cause of emergency room visits after motor vehicle collisions [2]. Whiplash is defined as a mechanism of energy transfer through the tissues of the neck in which some degree of tissue damage is likely. Their magnitude can range from a sprain/strain of soft tissues to complete rupture, joint dislocation, bony fracture, or even spinal cord trauma [3].

The annual incidence of WL is up to 70 per 100,000 individuals in Quebec, 106 per 100,000 individuals in Australia, 188–325 per 100,000 individuals in Holland, and 60.2 per 100,000 individuals in Spain. In these data, almost 90% of patients with WL registered in emergency room services exhibited evidence of grade II–III WL [4].

Whiplash injury (WL), understood as a cervical spine strain after trauma, can cause a great variety of clinical manifestations, such as pain and neck stiffness; numbness in the upper extremities; concentration and memory problems [5]; and other problems, like anxiety or depression [6]. However, there is no consensus regarding the multi-factorial mechanisms underlying prolonged WAD symptoms, perhaps because, despite progress in diagnosis through imaging tests, damage and physiological alterations are often not detectable. Some of the sequelae of WAD may be explainable as a mild traumatic brain injury; other theories include central sensitization, occult fracture, ligament instability, and changes in cervical lordosis [7,8,9]. Regarding this last hypothesis, although the association between changes in cervical lordosis and neck pain has been studied as a possible cause of cervical pain, there are not many studies on patients with whiplash injury [10,11].

In clinical practice, therapeutic exercise has shown evidence in reducing the duration and severity of WL in the treatment of pain, movement deficits, and disability, although this response may be variable, especially in the early stages [12]. There are several reviews with meta-analyses suggesting that exercise therapy lasting between 4 and 6 weeks may provide an additional effect for the improvement in neck pain and disability in patients with post-traumatic whiplash-associated disorders [13,14]. However, there is no consensus on which approach is best to manage these patients [15], and many reports reveal significant gaps in the information from studies investigating exercise for whiplash-associated disorders [16].

Moreover, high-velocity, low-amplitude manipulative techniques of the cervical spine seem to offer results in recent and persistent nonspecific neck pain without additional cost [17,18]. In fact, together with multimodal care, mobilization/manipulation has been recommended in clinical practice guidelines on the treatment of whiplash-associated disorders [19,20], combined with other treatments, such as educational videos and pain neuroscience education, exercises, or conventional physical rehabilitation [14,21]. All these treatments are also frequently used to treat neck problems, and they can produce similar changes in the short term, although with different healthcare costs. However, the optimum treatment and adequate dosage have not been determined, and there are insufficient data to assess long-term outcomes. Furthermore, although manipulative techniques seem to be effective in combination, there is insufficient evidence to confirm their benefit over more traditional techniques, as the available evidence on their effectiveness is limited, and the studies are of low quality [22]. Therefore, the main aim of the study was to determine the mid- and long-term efficiency (15, 30, and 120 days after starting sessions) of the cervical Specific Adjustment Technique (SAT) in patients with grade II acute WL, comparing it with a conventional rehabilitation program.

## 2. Materials and Methods

This prospective, single-blind, parallel-group randomized clinical trial was carried out in the physiotherapy service of the Clinic Hospital of Terrassa with the participation of the rehabilitation, radiology, traumatology, and surgery services from May 2024 to November 2024. The study was registered on ClinicalTrials.gov (NCT06389188). It was conducted in accordance with the Declaration of Helsinki and performed in accordance with relevant guidelines and regulations of the local Ethics Committee in Clinical Research of Clinic Hospital of Terrassa. The research is reported in accordance with the CONSORT guidelines. In addition, informed consent was obtained from all participants before data collection and interventions. Informed consent was also obtained to publish the images in an online open-access publication.

**Figure 1:**
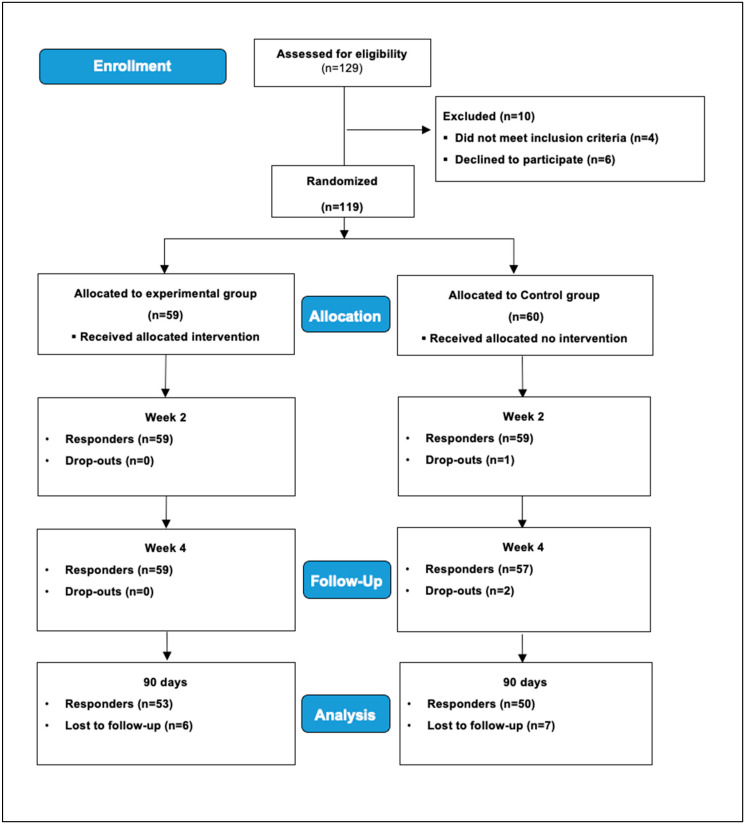
Flow-diagram of participants throughout the course of the study.

A total of 119 subjects with WL were recruited from emergency services of the Clinic Hospital of Terrassa. Prior to study, participants were randomly divided into two groups, MAN-Group (*n* = 59) and RHB-Group (*n* = 60), using the random number generator of a statistical program (SPSS version 24) to receive 3 and 20 treatment sessions, respectively (Figure 1). A single-blind study was performed because baseline measures were collected before randomization, and the outcome assessments were blinded. On the other hand, the patient did not know which procedure, experimental or control therapy, was going to be applied.

Inclusion criteria were patients between 18 and 60 years of age who went to the emergency service after suffering a traffic car collision and were the driver of the car, as well as diagnosed with acute WL grade II WAD by physicians from the traumatology service (with neck pain due to whiplash trauma with objective findings but no radiculopathy). In addition, patients had to be referred to the hospital rehabilitation service. The exclusion criteria include other symptomatology different from neck pain and other coexisting medical conditions, which could severely restrict participation in the study. The participants were asked not to seek other physiotherapy or pharmacological treatment during the study period.

**Figure 2:**
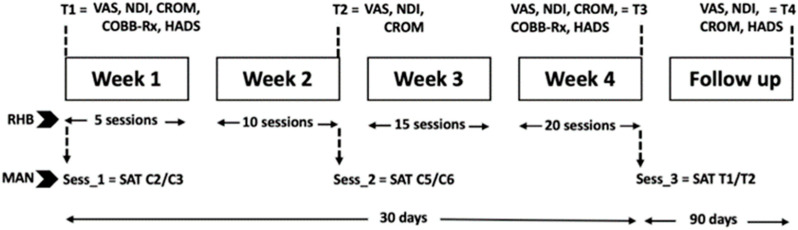
Sequence of treatment and outcome measures. VAS, Visual Analogic Scale; NDI, Neck Disability Index; CROM, Cervical Range of Motion; COOB-Rx, angular radiographic measurement using the Cobb method; HADS, hospital anxiety and depression scale; T1 to T4, first to fourth assessments; SAT, Specific Adjustment Technique in vertebral levels; Sess_1 to 3, sessions of treatment; MAN, experimental manipulation group; RHB, control rehabilitation group.

Pain with Visual Analogue Scale (VAS), Neck Disability Index (NDI), and Cervical Range of Motion (CROM) were measured at baseline, 15, 30, and 120 days after starting treatment sessions. Other outcomes were also measured at different times—hospital anxiety and depression scale (HADS) and cervical lordosis Cobb angle (COBB) (Figure 2).

### 2.1. Interventions

In the RHB group, the treatment protocol was carried out for four weeks, with five sessions per week, from Monday to Friday. Patients who were randomized into the MAN group were treated with cervical spine manipulation with SAT and received a total of 3 sessions of treatment over a month (days 1, 15, and 30 after the beginning of the study). Previously, a specialized physical therapist assessed that there was no risk of vertebro-basilar injuries following the International Framework [23]. Interventions in both groups occurred over the same time period, and all patients started their sessions during the first two weeks after the traffic car collision.

**Figure 3:**
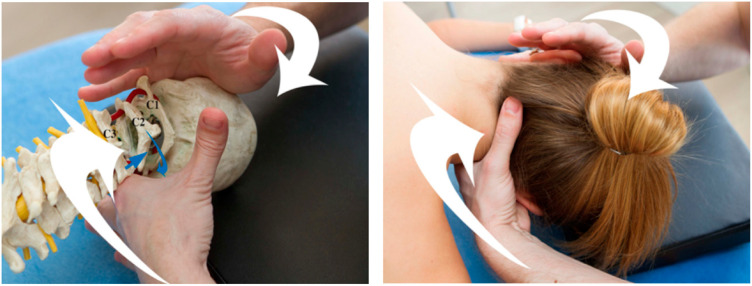
SAT technique C2 extension left rotation.

The SAT (see Video S1 in Supplementary Materials) was performed by a single physiotherapist with more than 20 years of experience in manual therapy. Treatment with SAT was applied over the superior cervical segment (cervical-2/cervical-3) after the baseline (T1) (Figure 3); the second intervention, over inferior cervical segment (cervical-5/cervical-6), was made after (T2); the third intervention was made after (T3), over superior thoracic segment (thoracic-1/thoracic-2). To ensure the safety of the treatment, there was a protocol in place to report and monitor adverse effects until the end of the study (T4), 90 days after the last intervention. In some cases, mild, short-term neurovegetative reactions (sweating or hypotension) appeared during or immediately after performing the treatment.

**Figure 4:**
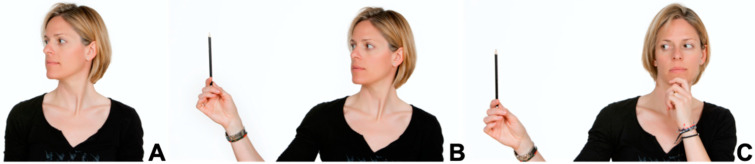
Ocular and cervical exercises. (**A**) Cervical exercises. Patient performs flexion and extension, left and right rotation, and side bending; (**B**) ocular exercises. Patient performs ocular movement in some directions without neck movement; (**C**) ocular and cervical exercises combined.

The RHB group was treated with passive manual therapy (P-MT) via soft tissue mobilization, massage, and muscular stretching of the anterior and posterior cervical muscles; active therapeutic exercises (A-TE); and oculo-cervical exercises (OC-E). Due to the high number of patients, a longer treatment time, and the limited resources available in the Rehabilitation Service of the Hospital de Terrassa, three different physiotherapists with more than 15 years of clinical experience participated. Before starting the study, they attended a 3-h workshop to reach a consensus on the techniques and their application. Patients received a total of 20 sessions of 30 min each over 4 weeks (Figure 2). The exercises were performed five times (30-s intervals each time) in a sitting position. Cervical movement was performed during expiration time, always in the same order. The eye exercise program was structured with cervical exercises, eye exercises, and a combination of both. Cervical exercises were performed in a seated position with an upright trunk supported by the chair backrest. During exhalation, the subjects executed cervical movements in a specific order: suboccipital flexion and extension, global cervical flexion and extension, right and left rotation, and right and left lateral flexion. Each cycle consisted of 5 repetitions, with a 30-s rest in a neutral position between cycles; ocular exercises were performed in a seated position with the trunk stabilized. The subjects held a reference object and directed their gaze in different directions (upward, downward, right, left, and diagonals) without moving the neck or trunk (Figure 4). Each cycle included 5 repetitions, followed by a 30-s rest to prevent dizziness or hyperventilation. To ensure proper execution, the subjects were advised to hold their chin with one hand to prevent compensatory movements; combination of exercises, both cervical and ocular exercises, were performed in the same sequence. The movement was always in the same order, finally describing diagonals. In addition, during the third and fourth weeks, the same previous protocol was performed, adding bilateral cervical stretching.

### 2.2. Outcome Measures

The baseline measures were collected before randomization, and the outcome assessments were blinded. All assessment measures, including range of motion and the Cobb angle on radiography, were collected by the same clinician, who was not involved in the treatment programs and did not know to which group belonged the outcomes assessed. Except for cervical range of motion, all outcome measures were recorded for statistical analysis only once. In addition, at the physiotherapy service, patients received a paper copy of the questionnaires to fill out on their own. As Figure 1 shows, four assessments were taken in this study in both groups (T1–T4).

### 2.3. Subjective Pain Intensity

Neck pain intensity was assessed with a Visual Analogue Scale (VAS) to measure the amount of pain experienced by a subject from both groups on a continuum from 0 to 100 mm. Scores can range from 0 (no pain) to 100 (worst imaginable pain). This method has been proven to be a reliable, generalizable, and internally consistent measure of clinical and experimental neck pain [24].

### 2.4. Neck Specific Disability

Neck-specific disability was measured with Neck Disability Index (NDI). The NDI is a valid measurement of disability in neck pain disorders. It is widely used, and it has shown good reliability and validity in WAD studies. NDI scores can range from 0% (no limitation on activity) to 100% (worst possible disability) [25,26].

### 2.5. Cervical Range of Motion (CROM)

Neck range of motion was tested with the CROM Instrument. The CROM attaches to the subject’s head and contains two gravity goniometers and one compass goniometer. All cervical motions and subsequent measurements were performed according to the manufacturer’s specifications and were reproduced exactly for each trial, with a single examiner performing all measurements [27]. CROM was assessed in a relaxed sitting position, hips and knees positioned at 90° angles and buttocks positioned against the back of the chair. The goniometer was placed on the top of the head and was set in the neutral position. The participants were instructed to hold at the end of the movement for three seconds for every register. Three measurements were recorded for each type of movement, and the mean was used in further statistical analysis. The CROM device is a reproducible measurement method for a symptomatic WAD population using the measurement protocol described [28].

### 2.6. Cervical Lordosis Cobb Angle

Cobb angle (COBB) method was measured to assess cervical lordosis as the angle between the horizontal line on the lower endplate of second cervical vertebra (C2) and a horizontal line on the lower endplate of seventh cervical vertebra (C7). A clinically normal cervical lordosis has been described as a Cobb angle of 31–40 degrees, with subjects standing and eyes focused straight ahead. A cervical lordosis of less than 20° from C2 to C7 has been shown to be related to cervical dysfunction and pain [9].

### 2.7. Hospital Anxiety and Depression Scale

Hospital anxiety and depression scale (HADS) was used to determine the levels of anxiety and depression experienced by participants. HADS has 14 items, and it was designed for the evaluation of anxiety and depression in non-psychiatric outpatient hospital services. The entire sum for both HADS anxiety and HADS depression levels ranges from 0 to 21. A score equal to or more than 10 indicates clinically significant symptoms of anxiety or depression [29].

### 2.8. Clinical Applications

This study aims to contribute to the advancement of the knowledge about the treatment of acute grade II WL, showing the effectiveness of SAT compared to other frequently used treatment protocols. The lack of randomized clinical trials related to the application of spinal manipulation techniques in whiplash injury, compared to studies in which passive and active interventions are applied, increases the interest in this study. According to the results, it could be included as part of used treatment protocols in the treatment of acute grade II WL since it could reduce the number of treatment sessions and associated healthcare costs.

### 2.9. Statistical Analysis

Sample size calculation with ANOVA of repeated measures of two ways was performed with the G*Power 3.1.9.2 software, with 0.25 medium effect size and at 5% significance level, power of 0.8; the sample size was *n* = 98 for both groups. We considered a sample loss of 20%, and 59 participants were required in each group. The main analyses were made on an intention-to-treat basis, including all available patients at either time point.

Data were analyzed with SPSS Version 24. Descriptive statistics were calculated, and normality of quantitative measures was verified. Finally, to study the main objective, mixed models of analysis of variance were separately estimated for each response. The between-subject factor was the treatment group, the intra-subject factor was the time of measurement, and the interaction between both was also analyzed. Age and gender were added as adjustment terms for the between-subject comparison. In cases of significant interaction, an additional simple effect analysis was performed. Cohen’s d effect size was calculated for each contrast. Absolute values of d were interpreted according to the outcome: a null effect for values < 0.20, a small effect for values of 0.20–0.50, a medium effect for values of 0.50–0.80, and a large effect for values > 0.80.

## 3. Results

One hundred and twenty-nine participants were screened for the eligibility criteria. One hundred and nineteen participants met the inclusion criteria and were randomly assigned to the MAN (*n* = 59) or RHB (*n* = 60) group to receive 3 and 20 treatment sessions over 4 weeks. At three-month follow-up, analyses were conducted in 53 participants of the experimental group and 50 participants of the control group. Table 1 shows descriptions of demographic and clinical data at first measurement for the entire sample and separated by groups. There were no significant differences between the groups before treatment or at follow-up for any outcomes. Table 2 shows the mean outcome for the two groups along the three follow-ups and the significance of the three effects analyzed in each mixed model. Table 3 shows the contrasts associated with the main effects of treatment and time for the previous mixed models. Regarding the interaction analysis for lordosis, Table 4 shows the simple effect analysis comparing the treatment effect for each measurement time for responses with significant interactions.

According to the type of treatment, statistically significant differences were found in flexion (*p* = 0.041) and left side bending (*p* = 0.022) in both cases, with the highest means in the experimental group. According to the effects of the time factor, all the outcomes showed significant general changes over time, which resulted in significant comparisons between each time point versus the baseline for all outcomes. Differences between time assessments were negative (pain, disability, anxiety, and depression) or positive (the rest of the measures) but reflected an improvement in the evaluation in all cases.

According to the interaction treatment–time effect, it was found that the evolution in both groups had statistically similar values in all measures except for the Cobb angle, where statistical significance was obtained (*p* = 0.047). The change between assessments after treatment and at baseline was positive for both treatment groups but only significant in the MAN group.

## 4. Discussion

The present study showed the effectiveness of SAT in grade II WAD treatment (neck pain and musculoskeletal signs) compared to a protocol based on therapeutic exercises and soft tissue mobilization. As hypothesized, the effects of SAT on pain, functionality, and CROM and the effects on anxiety and depression were comparable to other physiotherapy techniques [30]. However, the limited number of high-quality trials in this field limits the strength of the conclusions.

Initial outcome measures within the first 6 weeks after the accident in the acute phase of whiplash-associated disorder (WAD) were justified by the importance of measuring the initial response to the intervention, since improvements or changes are normally expected during this period [31]. This allows capturing the short-term effects of the intervention. Furthermore, it should be noted that the 30-day treatment period was established in the treatment protocol of the Rehabilitation Service of the Hospital de Terrassa. Finally, the 90-day follow-up period was included to compare, in both groups, the results in the acute management of WADII in the mid- and long-term since some patients may experience persistent symptoms. In this sense, there are studies that conclude that conservative and active interventions can be useful for reducing pain in the acute management of WADII in this period [4], but there are no studies on techniques such as SAT, which can offer comparable effects with a lower number of associated sessions.

In our study, we found that 3 treatment sessions in the experimental group were more effective than 20 treatment sessions in the control group. It is important to emphasize that the intervention in the experimental group lasted considerably less time (an average time of 45 min per session compared to 15 min for cervical manipulation). Although there is a knowledge gap in health economic evidence for non-invasive interventions in whiplash injuries, previous studies have been carried out to analyze not only the efficacy but also the cost-effectiveness relationship associated with different treatment approaches, like a behavioral therapy protocol or a manual therapy protocol compared with exercise or compared to a standard and conservative care program for people with neck pain following traffic accidents [32].

Because head orientation in space and posture require the visual, vestibular, and proprioceptive systems, neck and eye exercises are important in maintaining neck dynamics, and eye exercises have also been shown to influence cervical mobility. In addition, the mobilization of soft tissues, with massage and stretching of the anterior and posterior muscles of the neck, has been frequently applied, although caution is needed when drawing a valid conclusion on the efficacy of active and passive interventions in patients with whiplash injury [33].

De Rosario et al. [34] concluded that neck motion analysis was a useful objective tool to estimate part of the course of pain-related disability in WAD patients during the first months of rehabilitation. In this sense, Rahnama et al. [35] used dynamic ultrasound imaging to measure muscle deformation (elongation or shortening of the muscle during real-time movement) and the deformation rate (how fast the deformation occurs) of the deep dorsal neck muscles during a neck extension task. This research showed altered values associated with pain, disability, and fatigue between individuals with WAD and healthy controls. These findings possibly reflect that these muscles utilize altered strategies while performing a neck extension task. Indeed, adaptive dysfunctional patterns may develop that are secondary to the functional deficits. Peterson et al. [36] showed how three months of neck-specific exercises significantly improved ventral neck muscle interactions compared with staying on a waiting list. In our study, the differences obtained between the RHB group and the MAN group on CROM, only in flexion and left side bending, are difficult to explain. Despite this, improvement in CROM in the second measurement in both groups stands out, especially in the long-term measurement, where the greatest differences were obtained. Although more studies to verify these results are needed, the relevance of performing specific neck, scapulothoracic, and shoulder mobilization and strengthening exercises in the acute phase is raised [13].

Apart from that, considering that the association between cervical lordosis (sagittal alignment) and neck pain is controversial, some studies have proposed that decreased cervical lordosis, especially involving a kyphotic deformity from trauma, degeneration, or forward head posture, could cause neck pain [37]. For this reason, one of the short-term objectives should be to recover the curvature in the cervical lordosis, but it is unclear whether spinal manipulative therapy can change cervical lordosis since several investigations have obtained conflicting results [10,11]. One of the objectives of this study was to increase knowledge in this field and to determine whether cervical lordosis changes after three spinal manipulations in patients with grade II whiplash-associated disorders.

Although the RHB group had higher baseline cervical lordosis than the MAN group (22 vs. 18.1), there were no significant differences between the groups, and the final lordosis was practically the same (25.2 vs. 24.3). The treatments increased the Cobb angle in both groups (RHB = 3.24, MAN = 6.12), as reflected in the significant treatment by time interaction (*p* = 0.037), but only the SAT produced a significant increase (*p* = 0.047). Although there are few previous studies that are related, Harrison et al. [10] compared the effects of a sequence of cervical manipulations in a chronic cervicogenic pain group with a control group. Pretreatment and post-treatment Visual Analogue Scale (VAS) pain ratings and lateral cervical radiographs were analyzed for changes in alignment. This protocol decreased neck pain intensity and improved cervical lordosis. Anterior head weight-bearing was reduced by 11 mm, Cobb angles averaged an increase of 13 degrees, and the angle of intersection of posterior tangents on C2 and C7 averaged 17.9 degrees of improvement. These results are in line with those obtained in our study (an increase of 6.12 degrees in Cobb angle), considering that the differences obtained in the measurements may be the consequence of a greater number of interventions (3 manipulations over 4 weeks in our study vs. 38 manipulations over 15 weeks).

Although there is no strong relationship between cervical lordosis measurements, disability, and pain level [38], some studies, such as that by Yip et al. [39], related to the craniovertebral angle (CV) (the angle formed between a horizontal line through the spinous process of C7 and a line from the spinous process of C7 through the tragus of the ear, used to assess the posture of the head forward)), found a clear association of this variable with the degree of neck pain and disability.

Musculoskeletal disorders, especially WL, and myofascial pain syndromes may play an important role in the correlation between forward head posture, neck pain, and reduced cervical lordosis. In this sense, an increase in cervical lordosis, as indicated by Sun et al. (2014), could reduce the compression of the cervical facet joint and produce a decrease in pain [37].

The available studies that have reported a possible association between neck pain and the angle of lordosis have been carried out in different conditions of neck pain, but there are few studies with this objective in WL [40,41]. However, our findings reinforce the hypothesis that an increase in cervical lordosis could also be associated with a decrease in pain in patients with acute whiplash and would justify the use of spinal manipulation techniques (cervical and thoracic) in their treatment, as described in several systematic reviews on neck pain [3,13]. Additional studies are needed to clarify whether the CV or the Cobb angle could provide clinicians with more objective information about disability and severity in patients with neck pain [9].

Finally, psychological factors are suggested to be as important as collision severity in predicting the complaints and the changes related to treatment in collision victims with WAD grade I and II [42]. In our study, the results of anxiety and depression obtained with the HADS questionnaire show a greater decrease in the experimental group, although not significant, comparing measurement times T3 and T4 versus T1. These results agree with Wenzel et al. (2002), who found a positive association between whiplash injuries and anxiety and depression disorder (HADS questionnaire) in whiplash injuries produced more than 2 years ago but not in more recent whiplash injuries [43]. Part of the association between whiplash injuries and long-term anxiety and depression may be due to the personal wear and tear experienced by the patient when he perceives that the symptoms (mainly neck pain and headache) do not improve.

Before concluding, we need to address the limitations of this study. The large volume of patients, a longer treatment time, and limited resources available caused three different therapists to be assigned to the RHB group. This could increase the inter-researcher variations, although they had a previous meeting to reach a consensus. All the outcome assessments were taken by the same physician in all the variables. However, the radiologist who obtained the image could be different, although we also had a previous meeting at the baseline.

Additional limitations included the difficulty of blinding therapists and patients due to the type of manipulation technique and the number of lost-to-follow-up participants compared to those who dropped out during intervention. Regarding those lost to follow-up, it is difficult to have a broad, detailed understanding of the biopsychosocial and contextual issues influencing recovery and compliance with treatment [44]. It would have been interesting to implement some type of complementary intervention, such as educational meetings, to maintain the patient’s interest and adherence [45]. The possibility that the patient improved significantly could have influenced the results (a period between 30 and 130 days is estimated for recovering from pain) [46]. In our study, from the end of treatment until the last evaluation (T3 to T4 measurement), 90 days had passed (and 120 days since baseline).

We have not found studies that relate the loss rate or the lack of adherence with self-reported clinical improvement after a whiplash injury, but it is an interesting aspect that should be studied because it is possible that they are related. On the other hand, the payment of the financial compensation from the insurance company could be related since several studies have analyzed the influence of the economic factor in the recovery process [46].

## 5. Conclusions

The absence of significant differences between groups, before treatment and at follow-up, for any outcome makes the SAT comparable, and even better in some regard, to the treatments when treating pain and improving the functionality and mobility of the cervical spine. It was also successful in determining the evolution of states of anxiety and depression. Furthermore, it was shown to be effective when we found a loss of cervical lordosis since the significant treatment–time interaction showed that the Cobb angle increased in both groups, although only SAT produced a significant increase.

Although more research is needed on the effects of cervical manipulation techniques in grade II WL in the acute phase, due to its effectiveness and low associated healthcare cost (a shorter treatment period of 3 vs. 20 treatment sessions), SAT could be included in treatment protocols or clinical practice guidelines.

## Figures and Tables

**Table 1:** Baseline characteristics of participants.

	Total	MAN	RHB	*p*
Age	32.3 ± 9.3	30.6 ± 8.3	34.0 ± 10.4	0.212
Gender				0.315
Male	66 (55.5)	32 (53.6)	34 (56.7)	
Female	53 (44.5)	27 (46.4)	26 (43.3)	
Type of injury				0.886
Extension	8 (10.1)	5 (11.9)	3 (8.3)	
Flexion	70 (89.9)	37 (88.1)	33 (91.7)	
Employment				0.506
Yes	70 (58.8)	36 (61.0)	33 (55.0)	
No	49 (41.2)	23 (39.0)	27 (45.0)	
Days to start of treatment	18.1 ± 8.4	17.5 ± 6.1	18.7 ± 10.7	0.334
Pain (VAS)	6.5 ± 2.0	6.7 ± 2.0	6.6 ± 1.9	0.344
Disability (NDI)	46.5 ± 16.9	45.5 ± 16.1	47.6 ± 17.8	0.250
Cervical mobility (CROM) (°)				
Flexion	31.6 ± 13.9	31.7 ± 15.8	31.5 ± 11.9	0.472
Extension	41.6 ± 16.9	41.9 ± 17.9	41.3 ± 16.1	0.424
Right rotation	43.8 ± 16.4	44.6 ± 17.4	43.0 ± 15.5	0.293
Left rotation	42.5 ± 14.1	41.9 ± 15.1	43.2 ± 13.2	0.314
Right side bending	27.9 ± 10.5	28.2 ± 11.9	27.7 ± 9.0	0.394
Left side bending	30.8 ± 9.8	31.5 ± 10.2	30.1 ± 9.5	0.218
Anxiety (HADS)	10.3 ± 4.4	10.1 ± 4.1	10.5 ± 4.8	0.312
Depression (HADS)	7.2 ± 4.2	6.8 ± 3.6	7.6 ± 4.8	0.173
Lordosis (COOB)	20.0 ± 12.6	18.1 ± 11.9	22.0 ± 13.1	0.101

Values are mean ± SD or *n* (%). MAN, experimental manipulation group; RHB, control rehabilitation group; VAS, pain Visual Analogue Scale; NDI, Neck Disability Index; CROM, Cervical Range of Motion Instrument; HADS, hospital anxiety and depression scale; COOB, angular radiographic measurement using the Cobb method.

**Table 2:** Descriptive of outcomes and significance of components of mixed models.

	Mean					Treatment		Time		Treatment × Time	
	Treatment	T1	T2	T3	T4	*F*	*p*	*F*	*p*	*F*	*p*
Pain (VAS)	RHB	6.55	5.46	3.85	2.43	0.15	0.701	50.09	<0.005	0.73	0.774
	MAN	6.69	5.76	3.58	2.05						
Disability (NDI)	RHB	47.6	37.7	23.0	16.2	0.10	0.750	57.80	<0.005	0.31	0.817
	MAN	45.5	37.4	24.4	13.1						
Flexion (CROM)	RHB	31.5	32.9	39.5	44	4.22	0.041	18.85	<0.005	1.38	0.253
	MAN	31.7	39.1	46.5	48.9						
Extension (CROM)	RHB	41.3	48.6	52.1	60.5	0.03	0.851	22.43	<0.005	0.45	0.721
	MAN	41.9	45.9	55.9	63.2						
Right rotation (CROM)	RHB	43.0	48.3	53.4	59.5	2.87	0.092	20.75	<0.005	0.44	0.724
	MAN	44.6	52.2	58.7	60.4						
Left rotation (CROM)	RHB	43.2	48.9	58.2	61.1	0.27	0.604	32.69	<0.005	0.32	0.811
	MAN	41.9	50.9	59.4	59.9						
Right side bending (CROM)	RHB	27.6	31.9	34.0	37.2	2.92	0.089	20.03	<0.005	1.41	0.243
	MAN	28.2	33.4	39.8	40.4						
Left side bending (CROM)	RHB	30.1	34.4	35.6	39.5	5.31	0.022	22.19	<0.005	2.28	0.083
	MAN	31.5	37.2	43.3	44.6						
Lordosis (COOB)	RHB	22.0	NA	25.2	NA	1.64	0.202	4.42	0.037	0.42	0.037
	MAN	18.1		24.3							
Anxiety (HADS)	RHB	10.5	NA	7.4	7.0	0.11	0.736	21.02	<0.005	1.10	0.337
	MAN	10.1		7.3	5.0						
Depression (HADS)	RHB	7.6	NA	4.0	3.7	0.13	0.715	25.77	<0.005	1.11	0.333
	MAN	6.8		4.3	2.3						

Treatment, effect of treatment; Time, effect of time; Treatment × Time, interaction effect treatment by time; T1 to T4, first to fourth assessments; VAS, pain Visual Analogue Scale; NDI, Neck Disability Index; CROM, Cervical Range Of Motion Instrument; COOB, angular radiographic measurement using the Cobb method; HADS, hospital anxiety and depression scale; RHB, control rehabilitation group; MAN, experimental manipulation group; NA, not applicable; adjusted by age and gender.

**Table 3:** Contrasts between treatment groups and assessment times.

	MAN vs. RHB			T2 vs. T1			T3 vs. T1			T4 vs. T1		
	MD	*p*/*d*	95% CI	MD	*p*/*d*	95% CI	MD	*p*/*d*	95% CI	MD	*p*/*d*	95% CI
Pain (VAS)	0.11	0.701/0.04	−0.47; 0.70	−1.00	0.001/0.31	−1.58; −0.42	−2.90	<0.005/0.73	−3.63; −2.18	−4.38	<0.005/0.99	−5.19; −3.57
Disability (NDI)	0.64	0.750/0.03	−3.28; 4.55	−8.95	<0.005/0.34	−13.63; −4.26	−22.84	<0.005/0.87	−27.57; −18.11	−32.1	<0.005/1.06	−37.56; −26.56
Flexion (CROM)	3.36	0.041/0.19	0.14; 6.59	4.30	0.025/0.21	0.55; 8.05	11.34	<0.005/0.53	7.49; 15.20	14.95	<0.005/0.58	10.24; 19.67
Extension (CROM)	0.36	0.851/0.02	−3.41; 4.13	5.61	0.013/0.23	1.18; 10.05	13.40	<0.005/0.53	8.84; 17.95	20.30	<0.005/0.67	14.78; 25.81
Right rotation (CROM)	2.69	0.092/0.16	−0.44; 5.81	6.46	0.002/0.29	2.42; 10.51	12.24	<0.005/0.55	8.18; 16.29	16.27	<0.005/0.66	11.78; 20.75
Left rotation (CROM)	−0.84	0.604/0.05	−4.01; 2.34	7.26	<0.005/0.34	3.43; 11.10	16.20	<0.005/0.77	12.38; 20.02	18.02	<0.005/0.73	13.51; 22.53
Right side bending (CROM)	2.03	0.089/0.16	−0.31; 4.38	4.71	0.001/0.31	1.93; 7.50	8.95	<0.005/0.57	6.10; 11.80	11.20	<0.005/0.61	7.83; 14.60
Left side bending (CROM)	2.69	0.022/0.21	0.39; 4.99	4.93	<0.005/0.33	2.20; 7.65	8.54	<0.005/0.56	5.78; 11.30	11.67	<0.005/0.66	8.45; 14.90
Lordosis (COOB)	−3.04	0.202/0.12	−7.72; 1.65	NA			Table 4			NA		
Anxiety (HADS)	−0.21	0.736/0.03	−1.43; 1.01	NA			−2.92	<0.005/0.42	−4.18; −1.66	−4.34	<0.005/0.54	−5.80; −2.88
Depression (HADS)	−0.20	0.715/0.03	−1.26; 0.87	NA			−2.98	<0.005/0.48	−4.12; −1.85	−4.28	<0.005/0.61	−5.56; −3.00

MD, mean difference; MAN, experimental manipulation group; RHB, control rehabilitation group; T1; T4, first; fourth assessments; VAS, pain Visual Analogue Scale; NDI, Neck Disability Index; CROM, Cervical Range Of Motion Instrument; COOB, angular radiographic measurement using the Cobb method; HADS, hospital anxiety and depression scale; NA, Not applicable; *d*: Cohen’s d effect size; adjusted by age and gender.

**Table 4:** Interaction analysis for lordosis (COOB) for T3 vs. T1.

Treatment	T3 vs. T1		
	MD	*p*/*d*	95% CI
RHB	3.24	0.319/0.09	−3.16; 9.65
MAN	6.12	0.047/0.18	0.07; 12.17

MD, mean difference; COOB, angular radiographic measurement using the Cobb method; T3-T1, third and first assessments; RHB, control rehabilitation group; MAN, experimental manipulation group; *d*: Cohen’s d effect size; adjusted by age and gender.

## Data Availability

The data used in this study are available upon request. To access the dataset, interested parties must justify the need for its use and contact the corresponding author. Access will be subject to ethical and regulatory considerations and will be granted only for legitimate research purposes.

## Supplementary Materials

The following supporting information can be downloaded at: https://www.mdpi.com/article/10.3390/healthcare13070710/s1, CONSORT 2010 Checklist. The following supporting information can be downloaded at: https://drive.google.com/file/d/12zVZA2ujdWCb-VBGs_FGQdbh74CfRKNp/view?usp=sharing (accessed on: 3 February 2025), Video S1: Specific Adjustment Technique (SAT) in practice.

## Abbreviations

The following abbreviations are used in this manuscript:

WLs	Whiplash injuries.
SAT	Specific Adjustment Technique.
MAN	Manipulation group.
RHB	Rehabilitation group.
WAD	Whiplash Associated Disorders.
SPSS	Statistical Package for Social Sciences.
VAS	Visual Analogue Scale.
NDI	Neck Disability Index.
CROM	Cervical Range of Motion.
HADS	Hospital anxiety and depression scale.
COBB	Cobb angle.
COBB Rx	Cobb angle radiograph.
T1	Baseline.
T2	Second intervention.
T3	Third intervention.
T4	Fourth intervention.
P-MT	Passive manual therapy.
A-TEs	Active therapeutic exercises.
OC-Es	Oculo-cervical exercises.
C2	Second cervical vertebra.
C7	Seventh cervical vertebra.
Sess.	Session.
SAT C2/C3	Specific Adjustment Technique in the C2/C3 vertebral segment.
SAT C5/C6	Specific Adjustment Technique in the C5/C6 vertebral segment.
SAT T1/T2	Specific Adjustment Technique in the T1/T2 vertebral segment.

## References

[1] Tanaka N., Atesok K., Nakanishi K., Kamei N., Nakamae T., Kotaka S., Adachi N. (2018). Pathology and Treatment of Traumatic Cervical Spine Syndrome: Whiplash Injury. Adv. Orthop..

[2] Vattipally V.N., Weber-Levine C., Jiang K., Bhimreddy M., Kramer P., Davidar A.D., Hersh A.M., Winkle M., Byrne J.P., Azad T.D. (2024). Motor vehicle collision characteristics and hospitalization outcomes associated with mild traumatic brain injury and concomitant whiplash injury. Neurosurg. Focus.

[3] Wong J.J., Shearer H.M., Mior S., Jacobs C., Côté P., Randhawa K., Yu H., Southerst D., Varatharajan S., Sutton D. (2016). Are manual therapies, passive physical modalities, or acupuncture effective for the management of patients with whiplash-associated disorders or neck pain and associated disorders? An update of the Bone and Joint Decade Task Force on Neck Pain and Its Associated Disorders by the OPTIMa collaboration. Spine J..

[4] Wiangkham T., Duda J., Haque S., Madi M., Rushton A. (2015). The effectiveness of conservative management for acute whiplash associated disorder (WAD) II: A systematic review and meta-analysis of randomised controlled trials. PLoS ONE.

[5] Sarkilahti N., Leino S., Takatalo J., Loyttyniemi E., Tenovuo O. (2024). The symptom profile of people with whiplash-associated—A mixed method systematic review. J. Bodyw. Mov. Ther..

[6] Al-Khazali H.M., Ashina H., Iljazi A., Al-Sayegh Z., Lipton R.B., Ashina M., Ashina S., Schytz H.W. (2022). Psychiatric Sequelae Following Whiplash Injury: A Systematic Review. Front. Psychiatry.

[7] Elkin B.S., Elliott J.M., Siegmund G.P. (2016). Whiplash injury or concussion? A possible biomechanical explanation for concussion symptoms in some individuals following a rear-end collision. J. Orthop. Sports Phys. Ther..

[8] Anarte-Lazo E., Barbero M., Bernal-Utrera C., Rodríguez-Balnco C., Falla D. (2024). The association between neuropathic pain features and central sensitization with acute headache associated to a whiplash injury. Musculoskelet. Sci. Pract..

[9] Lee H.L., Jeon D.G., Park J.H. (2021). Correlation between kinematic sagittal parameters of the cervical lordosis or head posture and disc degeneration in patients with posterior neck pain. Open Med..

[10] Harrison D.E., Harrison D.D., Betz J.J., Janik T.J., Holland B., Colloca C.J., Haas J.W. (2003). Increasing the cervical lordosis with chiropractic biophysics seated combined extension-compression and transverse load cervical traction with cervical manipulation: Nonrandomized clinical control trial. J. Manip. Physiol. Ther..

[11] Shilton M., Branney J., de Vries B.P., Breen A.C. (2015). Does cervical lordosis change after spinal manipulation for non-specific neck pain? A prospective cohort study. Chiropr. Man. Ther..

[12] Southerst D., Nordin M.C., Côté P., Shearer H.M., Varatharajan S., Yu H., Wong J.J., Sutton D.A., Randhawa K.A., van der Velde G.M. (2016). Is exercise effective for the management of neck pain and associated disorders or whiplash-associated disorders? A systematic review by the Ontario Protocol for Traffic Injury Management (OPTIMa) Collaboration. Spine J..

[13] Chrcanovic B., Larsson J., Malmström E., Westergren H., Häggman-Henrikson B. (2021). Exercise therapy for whiplash-associated disorders: A systematic review and meta-analysis. Scand. J. Pain..

[14] Muñoz Lázcano P., Rojan Ortega D., Fernández López I. (2024). Effects of a Guided Neck-Specific Exercise Therapy on Recovery After a Whiplash: A Systematic Review and Meta-analysis. Am. J. Phys. Med. Rehabil..

[15] Castaldo M., Catena A., Chiarotto A., Fernández de las Peñas C., Arendt-Nielsen L. (2017). Do Subjects with Whiplash-Associated Disorders Respond Differently in the Short-Term to Manual Therapy and Exercise than Those with Mechanical Neck Pain?. Pain Med..

[16] Colombi A., Vedani S., Vicecont A., Stapleton C. (2024). The quality of reporting in randomized controlled trials investigating exercise for individuals with whiplash-associated disorders; a systematic review. Musculoskelet. Sci. Pract..

[17] García-González J., Romero-Del Rey R., Martinez-Martín V., Requena-Mullos M., Alarcón-Rodríguez R. (2024). Comparison of Short-Term Effects of Different Spinal Manipulations in Patients with Chronic Non-Specific Neck Pain: A Randomized Controlled Trial. Healthcare.

[18] Minnucci S., Innocenti T., Salvioli S., Giagio S., Yousif M.S., Riganelli F., Carletti C., Feller D., Brindisino F., Faletra A. (2023). Benefits and Harms of Spinal Manipulative Therapy for Treating Recent and Persistent Nonspecific Neck Pain: A Systematic Review With Meta-analysis. J. Orthop. Sports Phys. Ther..

[19] Bussières A.E., Stewart G., Al-Zoubi F., Decina P., Descarreaux M., Hayden J., Hendrickson B., Hincapié C., Pagé I., Passmore S. (2016). The Treatment of Neck Pain-Associated Disorders and Whiplash-Associated Disorders: A Clinical Practice Guideline. J. Manip. Physiol. Ther..

[20] Wong J.J., Côté P., Shearer H.M., Carroll L.J., Yu H., Varatharajan S., Southerst D., van der Velde G., Jacobs C., Taylor-Vaisey A. (2015). Clinical practice guidelines for the management of conditions related to traffic collisions: A systematic review by the OPTIMa Collaboration. Disabil. Rehabil..

[21] Willaert W., Leysen L., Lenoir D., Meeus M., Cagnie B., Nijs J., Sterling M., Coppieters I. (2021). Combining Stress Management With Pain Neuroscience Education and Exercise Therapy in People With Whiplash-Associated Disorders: A Clinical Perspective. Phys. Ther..

[22] Kim B.-J., Park A.-L., Hwang M.-S., Heo I., Park S.-Y., Cho J.-H., Kim K.-W., Lee J.-H., Ha I.-H., Park K.-S. (2022). Comparative Effectiveness and Safety of Concomitant Treatment with Chuna Manual Therapy and Usual Care for Whiplash Injuries: A Multicenter Randomized Controlled Trial. Int. J. Environ. Res. Public Health.

[23] Rushton A., Rivett D., Carlesso L., Flynn T., Hing W., Kerry R. (2014). International framework for examination of the cervical region for potential of cervical arterial dysfunction prior to orthopaedic manual therapy intervention. Man. Ther..

[24] Sterling M., Andersen T., Carroll L., Connelly L., Côté P., Curatolo M., Grant G., Jull G., Kasch H., Ravn S.L. (2023). Recommendations for a core outcome measurement set for clinical trials in whiplash associated disorders. Pain.

[25] Andrade-Ortega J.A., Delgado Martinez A.D., Almecija Ruiz R. (2008). Validation of a spanish version of the neck disability index. Med. Clin..

[26] Kovacs F.M., Bagó J., Royuela A., Seco J., Giménez S., Muriel A., Abraira V., Martín J.L., Peña J.L., Gestoso M. (2008). Psychometric characteristics of the spanish version of instruments to measure neck pain disability. BMC Musculoskelet. Disord..

[27] Audette I., Dumas J.P., Cote J.N., De Serres S.J. (2010). Validity and between-day reliability of the cervical range of motion (CROM) device. J. Orthop. Sports Phys. Ther..

[28] Williams M.A., Williamson E., Gates S., Cooke M.W. (2012). Reproducibility of the cervical range of motion (CROM) device for individuals with sub-acute whiplash associated disorders. Eur. Spine J..

[29] Lloyd M., Sugden N., Thomas M., McGrath A., Skilbeck C. (2023). The structure of the Hospital Anxiety and Depression Scale: Theoretical and methodological considerations. Br. J. Psychol..

[30] Teasell R.W., McClure J.A., Walton D., Pretty J., Salter K., Meyer M., Sequeira K., Death B. (2010). A research synthesis of therapeutic interventions for whiplash-associated disorder (WAD): Part 3—Interventions for subacute WAD. Pain Res. Manag..

[31] Alalawi A., Mazaheri M., Gallina A., Luque-Suarez A., Sterling M., Falla D. (2021). Are Measures of Physical Function of the Neck Region Associated With Poor Prognosis Following a Whiplash Trauma? A Systematic Review. Clin. J. Pain.

[32] van der Velde G., Yu H., Paulden M., Cote P., Varatharajan S., Shearer H.M., Wong J.J., Randhawa K., Southerst D., Mior S. (2016). Which interventions are cost-effective for the management of whiplash-associated and neck pain-associated disorders? A systematic review of the health economic literature by the Ontario protocol for traffic injury management (OPTIMa) collaboration. Spine J..

[33] Verhagen A.P., Scholten-Peeters G.M., van Wijngaarden S., de Bie R.A., Bierma-Zeinstra A. (2007). Conservative treatments for whiplash. Cochrane Database Syst. Rev..

[34] De Rosario H., Vivas M.J., Sinovas M.I., Page A. (2018). Relationship between neck motion and self-reported pain in patients with whiplash associated disorders during the acute phase. Musculoskelet. Sci. Pract..

[35] Rahnama L., Peterson G., Kazemnejad A., Trygg J., Peolsson A. (2018). Alterations in the mechanical response of deep dorsal neck muscles in individuals experiencing whiplash-associated disorders compared to healthy controls: An ultrasound study. Am. J. Phys. Med. Rehabil..

[36] Peterson G., Nilsson D., Trygg J., Peolsson A. (2018). Neck-specific exercise improves impaired interactions between ventral neck muscles in chronic whiplash: A randomized controlled ultrasound study. Sci. Rep..

[37] Sun A., Yeo H.G., Kim T.U., Hyun K.K., Kim J.Y. (2014). Radiologic assessment of forward head posture and its relation to myofascial pain syndrome. Ann. Rehabil. Med..

[38] Grob D., Frauenfelder H., Mannion A.F. (2007). The association between cervical spine curvature and neck pain. Eur. Spine J..

[39] Yip C.H., Chiu T.T., Poon A.T. (2008). The relationship between head posture and severity and disability of patients with neck pain. Man. Ther..

[40] Fortner M.O., Oakley P.A., Harrison D.E. (2018). Cervical extension traction as part of a multimodal rehabilitation program relieves whiplash-associated disorders in a patient having failed previous chiropractic treatment: A CBP^®^ case report. J. Phys. Ther. Sci..

[41] Norton T.C., Oakley P.A., Harrison D.E. (2023). Re-establishing the cervical lordosis after whiplash: A Chiropractic Biophysics^®^ spinal corrective care methods pre-auto injury and post-auto injury case report with follow-up. J. Phys. Ther. Sci..

[42] Daenen L., Nijs J., Raadsen B., Roussel N., Cras P., Dankaersts W. (2013). Cervical motor dysfunction and its predictive value for long-term recovery in patients with acute whiplash-associated disorders: A systematic review. J. Rehabil. Med..

[43] Wenzel H.G., Haug T.T., Mykletun A., Dahl A.A. (2002). A population study of anxiety and depression among persons who report whiplash traumas. J. Psychosom. Res..

[44] Stupar M., Côté P., Carroll L.J., Brison R.J., Boyle E., Shearer H.M., Cassidy J.D. (2023). Multivariable prediction models for the recovery of and claim closure related to post-collision neck pain and associated disorders. Chiropr. Man. Ther..

[45] Van der Wees P.J., Jamtvedt G., Rebbeck T., de Bie R.A., Dekker J., Hendriks E.J. (2008). Multifaceted strategies may increase implementation of physiotherapy clinical guidelines: A systematic review. Aust. J. Physiother..

[46] Boyle E., Cassidy J.D., Côte P., Carroll L.J. (2017). The relationship between insurance claim closure and recovery after traffic injuries for individuals with whiplash associated disorders. Disabil. Rehabil..

